# Effect of progesterone timing on live birth rates in day-6 blastocyst frozen-thawed embryo transfer cycles: a randomized controlled trial

**DOI:** 10.1093/hropen/hoag023

**Published:** 2026-03-16

**Authors:** Ruiqiong Zhou, Zhaoyi Wang, Mei Dong, Li Huang, Nianjun Su, Quan Qi, Ju Huang, Fuxiang Wang, Xiqian Zhang, Fenghua Liu

**Affiliations:** Centre for Reproductive Medicine, Guangdong Women and Children Hospital, Guangzhou, Guangdong, China; Women and Children’s Hospital, Southern University of Science and Technology, Shenzhen, Guangdong, China; Centre for Reproductive Medicine, Guangdong Women and Children Hospital, Guangzhou, Guangdong, China; Centre for Reproductive Medicine, Guangdong Women and Children Hospital, Guangzhou, Guangdong, China; Women and Children’s Hospital, Southern University of Science and Technology, Shenzhen, Guangdong, China; Centre for Reproductive Medicine, Guangdong Women and Children Hospital, Guangzhou, Guangdong, China; Women and Children’s Hospital, Southern University of Science and Technology, Shenzhen, Guangdong, China; Centre for Reproductive Medicine, Guangdong Women and Children Hospital, Guangzhou, Guangdong, China; Women and Children’s Hospital, Southern University of Science and Technology, Shenzhen, Guangdong, China; Centre for Reproductive Medicine, Guangdong Women and Children Hospital, Guangzhou, Guangdong, China; Women and Children’s Hospital, Southern University of Science and Technology, Shenzhen, Guangdong, China; Centre for Reproductive Medicine, Guangdong Women and Children Hospital, Guangzhou, Guangdong, China; Women and Children’s Hospital, Southern University of Science and Technology, Shenzhen, Guangdong, China; Centre for Reproductive Medicine, Guangdong Women and Children Hospital, Guangzhou, Guangdong, China; Women and Children’s Hospital, Southern University of Science and Technology, Shenzhen, Guangdong, China; Centre for Reproductive Medicine, Guangdong Women and Children Hospital, Guangzhou, Guangdong, China; Women and Children’s Hospital, Southern University of Science and Technology, Shenzhen, Guangdong, China; Centre for Reproductive Medicine, Guangdong Women and Children Hospital, Guangzhou, Guangdong, China; Women and Children’s Hospital, Southern University of Science and Technology, Shenzhen, Guangdong, China

**Keywords:** frozen-thawed embryo transfer, live birth rate, progesterone timing, day-6 blastocyst, blastocyst expansion stage, artificial cycle

## Abstract

**STUDY QUESTION:**

What is the optimal timing of progesterone exposure to achieve live births for day-6 blastocysts in artificial frozen-thawed embryo transfer (FET) cycles?

**SUMMARY ANSWER:**

Live birth rates (LBRs) were comparable between day-6 and day-7 progesterone supplementation for day-6 blastocyst transfers in artificial FET cycles.

**WHAT IS KNOWN ALREADY:**

In artificial FET cycles, embryo–endometrium synchrony is primarily determined by the duration of progesterone exposure. Although blastocyst transfer is conventionally performed on day six of progesterone supplementation, the optimal duration of progesterone exposure remains uncertain. Available evidence suggests that the window of implantation may be relatively flexible for day-5 blastocysts, whereas day-6 blastocysts may have a distinct and potentially narrower window of implantation. However, prospective data evaluating different durations of progesterone exposure for blastocyst transfer—particularly for day-6 blastocysts—remain limited.

**STUDY DESIGN, SIZE, DURATION:**

This open-label randomized controlled trial (RCT) included 338 women undergoing FET in artificial cycles who had exclusively day-6 blastocysts available for transfer at enrolment. These blastocysts originated either from IVF/ICSI cycles with exclusive day-6 blastocyst formation or from previous embryo transfer attempts in which only day-6 blastocysts remained cryopreserved. Participants were randomized prior to progesterone initiation to undergo blastocyst transfer on 6 or 7 days of progesterone supplementation. Recruitment occurred between September 2022 and May 2024, with final follow-up completed in March 2025.

**PARTICIPANTS/MATERIALS, SETTING, METHODS:**

A total of 338 participants were randomized equally to undergo blastocyst transfer on either Day 6 or Day 7 of progesterone supplementation. The primary outcome was LBR. Secondary outcomes included singleton and twin LBR, biochemical and clinical pregnancy, pregnancy loss, good birth outcome, and maternal and neonatal outcomes. The primary analysis followed the intention-to-treat (ITT) principle, with a per-protocol (PP) analysis also performed. Post hoc analyses included multivariable regression and subgroup analyses.

**MAIN RESULTS AND THE ROLE OF CHANCE:**

In the ITT analysis, the LBR was 45.0% (76/169) in the day-6 progesterone group and 40.2% (68/169) in the day-7 group (absolute difference, −4.7% [95% CI, −15.3% to 5.8%]; adjusted relative ratio (RR), 0.90 [95% CI, 0.70–1.14], *P *= 0.367). Rates of biochemical pregnancy, clinical pregnancy, pregnancy loss, and good birth outcome were comparable between groups. No differences were observed in maternal and neonatal outcomes. PP analysis yielded results consistent with the ITT analysis. Exploratory post hoc subgroup analyses identified a significant interaction between blastocyst expansion stage and treatment group (*P* for interaction = 0.004). Among early blastocyst transfers, the LBR was significantly lower in the day-7 progesterone group compared with the day-6 group (36.8% vs 53.8%; absolute difference, −17.1% [95% CI, −30.3% to −3.8%]; adjusted RR, 0.69 [95% CI, 0.51–0.94], *P *= 0.018). In contrast, among late blastocyst transfers, the LBR tended to be higher in the day-7 group (46.8% vs 31.7%; absolute difference, 15.0% [95% CI, −1.9% to 32.0%]; adjusted RR, 1.47 [95% CI, 0.95–2.28], *P *= 0.082), although this difference did not reach statistical significance.

**LIMITATIONS, REASONS FOR CAUTION:**

This study was open-label, which may have introduced performance bias; however, embryologists were blinded to group allocation, and all outcomes were objective, minimizing the risk of detection bias. Subgroup analyses were conducted post hoc, and the sample size may have been insufficient to detect differences within these subgroups. Accordingly, these findings should be interpreted with caution. In addition, the study was not powered to assess potential differences in treatment effects among specific subpopulations, such as patients with polycystic ovary syndrome, endometriosis, or repeated implantation failure.

**WIDER IMPLICATIONS OF THE FINDINGS:**

Based on this trial, LBRs were comparable between transfers performed on six versus seven days of progesterone exposure for day-6 blastocysts in artificial FET cycles. Exploratory subgroup findings, including those related to blastocyst expansion stage, are hypothesis-generating and require confirmation in future adequately powered RCTs.

**STUDY FUNDING/COMPETING INTEREST(S):**

The study was funded by the Natural Science Foundation of Guangdong Province, China (2025A1515010073). No conflicts of interest are declared for all authors.

**TRIAL REGISTRATION NUMBER:**

Chinese Clinical Trial Registry (No. ChiCTR2200062901). URL: https://www.chictr.org.cn/bin/project/edit?pid=175020.

**TRIAL REGISTRATION DATE:**

23 August 2022.

**DATE OF FIRST PATIENT’S ENROLMENT:**

12 September 2022.

WHAT DOES THIS MEAN FOR PATIENTS? This study explored the best timing of progesterone supplementation to improve live birth rates for frozen day-6 blastocysts. Progesterone prepares the womb for embryo implantation, but it was unclear whether transferring day-6 blastocysts on the sixth or seventh day of progesterone exposure would provide a better chance of having a baby.

We conducted a clinical trial involving 338 women who had only day-6 blastocysts available. Participants were randomly assigned to undergo embryo transfer on either the sixth or seventh day of progesterone supplementation. Overall, live birth rates were similar between the two groups. However, when we looked more closely, early-stage blastocysts (less developed) achieved higher live birth rates with six days of progesterone, while late-stage blastocysts (more developed) tended to do better with seven days.

These findings provide new insights into the optimal timing for day-6 blastocyst transfers and suggest that the duration of progesterone exposure before transfer may need to be tailored to the developmental stage of the blastocyst to improve pregnancy outcomes.

## Introduction

The use of frozen-thawed embryo transfer (FET) has increased substantially worldwide in recent decades, owing to advances in vitrification technology (Rienzi *et al.*, 2017). FET is now widely applied across a range of clinical indications, including the prevention of ovarian hyperstimulation syndrome, preimplantation genetic testing (PGT), management of premature progesterone elevation, optimization of embryo–endometrium synchrony, and fertility preservation (Shapiro *et al.*, 2014; Roque *et al.*, 2015, 2019; Healy *et al.*, 2016).

Several protocols have been proposed for endometrial preparation in FET cycles, including natural, ovarian stimulation, and HRT cycles; however, the optimal approach for endometrial preparation remains uncertain (Wu *et al.*, 2021; Ho *et al.*, 2024; Ghobara *et al.*, 2025; Liu *et al.*, 2025). Emerging evidence suggests that artificial FET cycles, which lack a functional corpus luteum, may be associated with a higher risk of adverse obstetric outcomes compared with natural or stimulated cycles (Zaat *et al.*, 2023; Bülow *et al.*, 2025). Despite this evolving landscape, HRT cycles remain widely used because of their scheduling flexibility and are indispensable in specific clinical settings, such as in women with premature ovarian insufficiency, postmenopausal women, and oocyte donation programmes.

Successful implantation depends on precise synchrony between embryonic development and endometrial receptivity (Paria *et al.*, 2002). In FET cycles, this synchrony is primarily determined by the duration of progesterone exposure, which defines the window of implantation (WOI) (Mackens *et al.*, 2017). Suboptimal timing of progesterone supplementation may result in embryo–endometrium dys-synchrony and compromised implantation potential (Franasiak *et al.*, 2016). Nevertheless, the optimal timing of embryo transfer after progesterone initiation has not been clearly established, especially in HRT cycles.

For frozen cleavage-stage embryo transfers, previous studies have reported inconsistent findings regarding the optimal progesterone exposure prior to FET (Nawroth and Ludwig, 2005; van de Vijver *et al.*, 2016; Glujovsky *et al.*, 2020). A randomized controlled trial (RCT) showed comparable pregnancy rates when vitrified–warmed cleavage-stage embryos were transferred after 3 or 5 days of progesterone administration; however, embryo transfer on the third day of progesterone exposure was associated with a significantly higher incidence of early pregnancy loss (van de Vijver *et al.*, 2016). To date, only one RCT has evaluated different durations of progesterone exposure before blastocyst transfer, reporting no significant difference in clinical pregnancy rates between transfers performed on the fifth versus seventh day of progesterone supplementation (van de Vijver *et al.*, 2017). Importantly, this study did not specifically address potential differences related to blastocyst developmental timing.

Embryonic developmental speed represents another critical determinant of embryo–endometrium synchrony. Compared with day-5 blastocysts, day-6 blastocysts exhibit delayed blastulation and have generally been associated with lower clinical pregnancy rates in both fresh and frozen cycles (Haas *et al.*, 2016; Ferreux *et al.*, 2018). In routine clinical practice, blastocyst transfer—regardless of developmental timing—is typically performed after a uniform duration of progesterone exposure, most commonly six days. Under this standard protocol, multiple studies have consistently reported inferior reproductive outcomes for day-6 blastocysts compared with day-5 blastocysts (Sunkara *et al.*, 2010; Haas *et al.*, 2016; Yang *et al.*, 2016; Ferreux *et al.*, 2018; Bourdon *et al.*, 2019).

Retrospective cohort studies have shown comparable LBRs between transfers performed on six versus seven days of progesterone supplementation in artificial FET cycles (Roelens *et al.*, 2020; Zhou *et al.*, 2024). However, stratified analyses by blastocyst developmental stage suggest that day-6 blastocysts transferred on six days of progesterone exposure are associated with less favourable pregnancy outcomes compared with those transferred on seven days, whereas outcomes for day-5 blastocysts appear similar between regimens. In current clinical practice, a substantial proportion of blastocysts reach the blastocyst stage on Day 6 rather than Day 5. Previous studies have reported that ∼25–45% of blastocysts transferred in FET cycles are day-6 blastocysts (Desai *et al.*, 2016; Roelens *et al.*, 2020; Zhou *et al.*, 2024), underscoring the clinical relevance of optimizing transfer strategies for this population.

To date, no prospective clinical trial has specifically evaluated the optimal duration of progesterone exposure in FET cycles involving exclusively day-6 blastocysts. Accordingly, we conducted an RCT to compare pregnancy outcomes between transfers performed on six versus seven days of progesterone supplementation.

## Materials and methods

### Study design

This open-label RCT was conducted at the Centre for Reproductive Medicine, Guangdong Women and Children Hospital. The study protocol was approved by the Ethics Committee of Guangdong Women and Children Hospital (Approval No. 202201166) and was registered in the Chinese Clinical Trial Registry on 23 August 2022 (ChiCTR2200062901). Written informed consent was obtained from all participants. The study was conducted in accordance with the Declaration of Helsinki and adhered to the principles of Good Clinical Practice.

### Participants

Eligible participants were women aged 20–40 years undergoing FET in artificial cycles who had exclusively day-6 blastocysts available for transfer at enrolment. This population comprised two clinical scenarios: (i) exclusive day-6 blastocyst formation in the IVF/ICSI cycle, and (ii) women with previous embryo transfer attempts in whom only day-6 blastocysts remained cryopreserved and available at enrolment. Exclusion criteria included a history of recurrent pregnancy loss (defined as ≥2 failed clinical pregnancies), more than three previous embryo transfer attempts, thin endometrium (<7 mm), uterine abnormalities (e.g. adenomyosis or intrauterine adhesions), oocyte donation cycles, escape ovulation (serum progesterone >1.0 ng/mL), GnRH agonist pre-treatment, or double-embryo transfer. Eligibility screening was conducted between menstrual cycle days 2 and 4 of the FET cycle, and all participants provided written informed consent after thorough counselling.

### Randomization and masking

Eligible participants were randomized on the day prior to the initiation of progesterone supplementation using a random sequence generated by SPSS software (version 22.0, IBM Corp., Armonk, NY, USA). Randomization was performed by an independent research assistant who was not involved in participant recruitment or clinical management. The research assistant delivered the treatment allocations to the research team in sealed envelopes. Participants were allocated in a 1:1 ratio to undergo blastocyst transfer on either Day 6 or Day 7 of progesterone supplementation. Given the obvious treatment differences between the two groups, the trial was open-label; however, embryologists assessing the embryos were blinded to group allocation.

### Procedures

FET was performed in HRT cycles. In HRT-FET cycles, a daily oestradiol dose of 4–6 mg is commonly used to achieve adequate endometrial proliferation; however, the optimal oestradiol dosage and administration pattern have not been definitively established (Mumusoglu *et al.*, 2021; Hizkiyahu *et al.*, 2022; Zhang *et al.*, 2023). At our centre, after confirming that patients were in the early proliferative phase of the menstrual cycle (absence of follicles ≥10 mm in diameter, endometrial thickness ≤6 mm, serum oestradiol <80 pg/mL, and serum progesterone <1 ng/mL), oral oestradiol valerate (Progynova; Bayer Schering Pharma AG, Berlin, Germany) was initiated on cycle days 2–4 at a dose of 2 mg twice daily for 5–6 days, followed by 3 mg twice daily for an additional 5–6 days. After ∼10 days of oestrogen administration, transvaginal ultrasound was performed to assess endometrial thickness and morphology, and serum levels of oestradiol, progesterone, and luteinizing hormone were measured. The dose and duration of oestradiol valerate were adjusted as appropriate according to endometrial and hormonal response. If endometrial thickness remained <7 mm and serum progesterone was < 1 ng/mL, oestradiol valerate was continued at 3 mg twice daily, with reassessment until a thickness of ≥ 7 mm was achieved. Once the endometrial thickness reached ≥7 mm, oestradiol was continued and luteal phase support was initiated the following morning with vaginal progesterone gel (Crinone, Merck Serono, Darmstadt, Germany) at 90 mg once daily combined with oral dydrogesterone (Duphaston, Abbott, Chicago, IL, USA) at 10 mg three times daily. Given the absence of a universally accepted luteal phase support protocol for HRT cycles, this combined regimen was used to ensure adequate progesterone exposure (Melo *et al.*, 2021, 2022; Toriumi *et al.*, 2023; Vidal *et al.*, 2023; Garg *et al.*, 2024). Day-6 blastocyst transfer was performed on either the sixth or seventh day of progesterone administration. If clinical pregnancy was confirmed, luteal phase support was continued until 10 weeks of gestation.

### Embryos evaluation and transfer procedure

Blastocysts were assessed for expansion, inner cell mass (ICM) quality, and trophectoderm (TE) appearance on Day 5 or 6 according to the Gardner and Schoolcraft grading system (Gardner *et al.*, 2002). Embryos graded ≥3BC were cryopreserved. Expansion was classified as follows: stage 3, full blastocyst with the blastocoel completely filling the embryo; stage 4, expanded blastocyst with the blastocoel enlarging the embryo and thinning the zona pellucida; stage 5, hatching blastocyst with the TE herniating through the zona pellucida; stage 6, fully hatched blastocyst. ICM morphology was graded as A (numerous tightly packed cells), B (several loosely grouped cells), or C (very few cells), and TE morphology was graded as A (many cells forming a cohesive epithelium), B (few cells forming a loose epithelium), or C (very few large cells). Blastocysts were thawed on the morning of embryo transfer, and post-warming survival was defined as ≥50% of blastomeres or blastocyst cells intact without degeneration. Morphology was reassessed after thawing. Good-quality blastocysts were defined as those with at least stage 3 expansion and both ICM and TE graded A or B (≥3BB). If the first thawed blastocyst did not survive, a second blastocyst was thawed and transferred.

### Outcomes

The primary outcome was the LBR, defined as the delivery of a live infant at ≥24 weeks of gestation. Secondary outcomes included singleton and twin LBR, biochemical pregnancy, clinical pregnancy, pregnancy loss, good birth outcome, gestational age at delivery, birth weight, and maternal and neonatal complications. Detailed definitions of all secondary outcomes are provided in Supplementary Table S1.

### Sample size

Based on a retrospective study, the LBR for day-6 blastocysts was lower in FET cycles performed on Day 6 of progesterone exposure compared with those on Day 7 (21.5% vs 35.5%) (Roelens *et al.*, 2020). Assuming a 14% absolute difference in LBR between the two randomized groups, the required sample size was calculated using PASS software (version 15.0.5, NCSS, Kaysville, UT, USA). With a two-sided significance level (α) of 0.05, a statistical power (1–β) of 80%, and an anticipated dropout rate of 5%, the final sample size was determined to be 169 participants per group, yielding a total of 338 participants.

### Statistical analysis

The primary analysis of all primary and secondary outcomes followed the intention-to-treat (ITT) principle, with all randomized participants analysed according to their assigned groups. Continuous variables that approximate a normal distribution are reported as mean ± SD and compared using independent-samples *t*-tests; non-normally distributed continuous variables are presented as median (range) and compared using the Mann–Whitney *U*-test. Categorical variables are presented as counts and percentages and compared using Pearson’s chi-square test or Fisher’s exact test when expected frequencies are <5. Relative ratios (RRs) with 95% CIs, together with absolute differences and their 95% CIs, are reported.

For secondary analyses, a per-protocol (PP) analysis was conducted, including only participants who fully adhered to the study protocol and excluding those with any protocol deviations. Post hoc analyses included multivariable regression models and subgroup analyses. In the ITT population, LBRs were compared between treatment groups using regression modelling. Adjusted RRs and 95% CIs were estimated using log-binomial regression; if model convergence failed, Poisson regression with robust SEs was applied. Subgroup analyses were conducted by age (<35 vs ≥35 years), blastocyst expansion stage (early vs late), BMI (<24 vs ≥24 kg/m^2^), type of infertility (primary vs secondary), number of IVF cycles (one vs ≥2), good-quality blastocyst transfer (no vs yes), and PGT cycle (no vs yes).

All hypothesis tests were two-sided, and a *P* value <0.05 was considered statistically significant. Analyses were conducted using SPSS (IBM Corp.) and R (version 4.3, R Foundation for Statistical Computing, Vienna, Austria).

## Results

### Study participants

**Figure 1.**
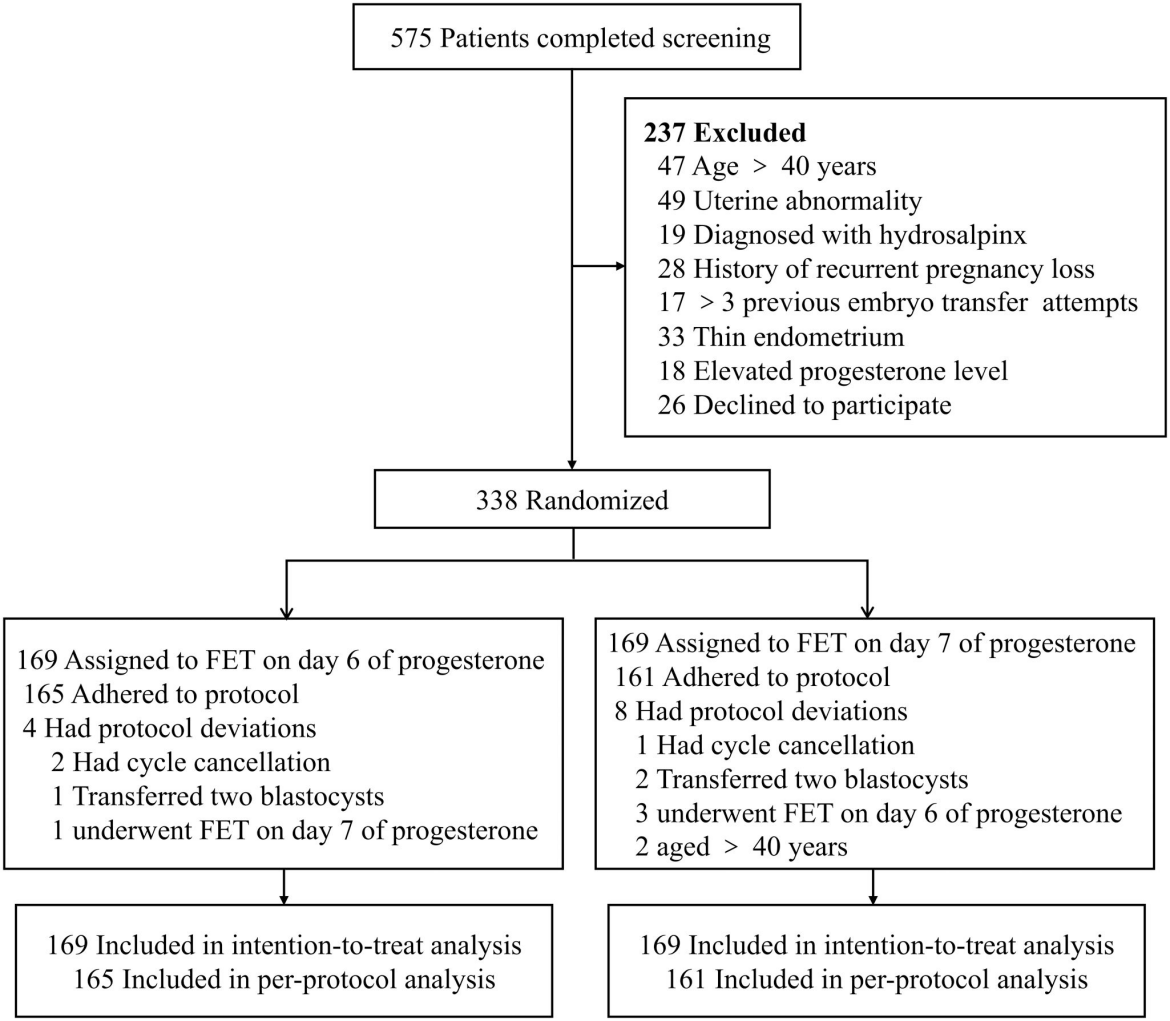
**Flowchart of patient enrolment, randomization, follow-up, and analysis in a randomized clinical trial evaluating the effect of progesterone timing on live birth rates in day-6 blastocyst frozen-thawed embryo transfer cycles**. FET, frozen-thawed embryo transfer.

Between September 2022 and May 2024, a total of 575 women were screened for eligibility, of whom 237 were excluded, and 338 participants were randomized equally into two groups (169 per group) (Fig. 1). Overall, 326 women (96.4%) adhered to the study protocol. Protocol deviations occurred in 4 of 169 (2.4%) women in the day-6 progesterone FET group and in 8 of 169 (4.7%) in the day-7 group (Table 1).

**Table 1.** Baseline, IVF cycle, and FET cycle characteristics in the intention-to-treat population.

Parameters	Day-6 progesterone group	Day-7 progesterone group
	(n = 169)	(n = 169)
**Baseline characteristics**		
Age at retrieval (years)	31.0 (28.0–34.0)	31.0 (28.0–34.5)
Age at randomization (years)	32.0 (30.0–35.0)	32.0 (29.0–36.0)
Body mass index (kg/m^2^)	21.6 (19.8–23.8)	21.6 (19.9–23.6)
Duration of infertility (years)	2.0 (1.0–4.3)	2.0 (1.0–4.5)
Type of infertility		
Primary	88 (52.1)	82 (48.5)
Secondary	81 (47.9)	87 (51.5)
Indications for IVF		
Tubal factor	62 (36.7)	68 (40.2)
Male factor	29 (17.2)	24 (14.2)
Others	54 (32.0)	53 (31.4)
Combined factors	14 (8.3)	14 (8.3)
Unexplained	10 (5.9)	10 (5.9)
Antral follicle count	15 (10–22)	15 (11–20)
Polycystic ovary syndrome	29 (17.2)	30 (17.8)
Repeated implantation failure	6 (3.6)	4 (2.4)
Endometriosis	13 (7.7)	11 (6.5)
**IVF cycle characteristics**		
Number of IVF cycles		
1	141 (83.4)	145 (85.8)
2	27 (16.0)	24 (14.2)
3	1 (0.6)	0
ICSI treatment	90 (53.3)	92 (54.4)
Number of oocytes retrieved	16 (12–22)	16 (12–22)
Number of good-quality embryos on Day 3	5 (3–7)	5 (3–7)
Number of blastocysts (D5 and D6)	4 (2–5)	4 (2–6)
Number of good-quality blastocysts (D5 and D6)	1 (0–2)	1 (0–3)
Without D5 blastocyst formation in fresh cycle	131 (77.5)	131 (77.5)
Number of D6 blastocysts	3 (2–5)	3 (2–5)
**FET cycle characteristics**		
Previous embryo transfer attempts	1 (0–1)	1 (0–1)
Endometrial thickness*	9.0 (8.0–10.5)	9.0 (8.0–11.0)
Serum progesterone before progesterone^#^	0.16 (0.09–0.24)	0.17 (0.09–0.28)
Serum estradiol before progesterone^#^	230.5 (177.1–296.4)	230.5 (185.9–320.9)
Days of estradiol before progesterone^#^	14 (13–17)	15 (13–17)
Good-quality blastocyst transfer	89 (53.3)	90 (53.6)
Two blastocysts	1 (0.6)	2 (1.2)
PGT cycle	54 (32.3)	55 (32.7)
Blastocyst expansion stage		
3	9 (5.4)	6 (3.6)
4	95 (56.9)	100 (59.5)
5	31 (18.6)	36 (21.4)
6	32 (19.2)	26 (15.5)
Protocol deviations	4 (2.4)	8 (4.7)

Data are median (interquartile range) or n (%). Continuous variables were compared using Mann–Whitney *U*-test; categorical variables were compared using the chi-square test or Fisher’s exact test. FET, frozen embryo transfer; PGT, preimplantation genetic testing; D5, day-5 blastocyst; D6, day-6 blastocyst.

* The day of frozen embryo transfer.

# The day before progesterone initiation. Repeated implantation failure was defined as failure to achieve a clinical pregnancy after ≥3 embryo transfer attempts, including the transfer of ≥2 good-quality blastocysts or ≥4 good-quality cleavage-stage embryos.

Baseline characteristics, as well as IVF and FET cycle parameters, were well balanced between the two randomized groups in the ITT population (Table 1). Per-protocol analyses excluding protocol deviations yielded results consistent with those of the ITT analysis (Supplementary Table S2).

### Primary outcome

In the ITT analysis, the LBR was 45.0% (76/169) in the day-6 progesterone group and 40.2% (68/169) in the day-7 group (absolute difference, −4.7% [95% CI, −15.3% to 5.8%]; adjusted RR, 0.90 [95% CI, 0.70–1.14]; *P *= 0.367; Table 2). The proportions of singleton and twin live births were comparable between groups. Per-protocol analysis yielded consistent results, with LBRs of 44.8% (74/165) for day-6 and 41.6% (67/161) for day-7 progesterone (absolute difference, −3.2%; 95% CI, −14.0% to 7.5%; adjusted RR, 0.93 [95% CI, 0.73–1.18]; *P *= 0.555; Supplementary Table S3).

**Table 2.** Live birth, pregnancy, and pregnancy loss in the intention-to-treat population.

Outcomes	Day-6 progesterone group	Day-7 progesterone group	Absolute difference	Unadjusted RR	Unadjusted	Adjusted RR	Adjusted
			(95% CI)	(95% CI)	*P* value	(95% CI)	*P* value
Primary outcome	n = 169	n = 169					
Total livebirth per women	76/169 (45.0)	68/169 (40.2)	−4.7 (−15.3 to 5.8)	0.89 (0.70–1.15)	0.379	0.90 (0.70–1.14)	0.367
Singleton livebirth per women	76/169 (45.0)	66/169 (39.1)	−5.9 (−16.4 to 4.6)	0.87 (0.68–1.12)	0.270	0.87 (0.68–1.11)	0.259
Twin livebirth per women	0	2/169 (1.2)	1.2 (−0.4 to 2.8)		0.499		
Biochemical pregnancy	105/169 (62.1)	97/169 (57.4)	−4.7 (−15.2 to 5.7)	0.92 (0.78–1.10)	0.375	0.92 (0.78–1.09)	0.315
Clinical pregnancy	92/169 (54.4)	83/169 (49.1)	−5.3 (−16.0 to 5.3)	0.90 (0.73–1.11)	0.327	0.90 (0.74–1.09)	0.282
Total pregnancy loss	28/105 (26.7)	29/97 (29.9)	3.2 (−9.2 to 15.7)	1.12 (0.72–1.74)	0.610	1.11 (0.71–1.71)	0.649
Biochemical pregnancy loss	13/105 (12.4)	14/97 (14.4)	2.1 (−7.4 to 11.5)	1.17 (0.58–2.35)	0.669	1.17 (0.57–2.39)	0.674
Clinical pregnancy loss	15/92 (16.3)	15/83 (18.1)	1.8 (−9.4 to 13.0)	1.11 (0.58–2.13)	0.757	1.09 (0.57–2.08)	0.796
First trimester pregnancy loss	10/92 (10.9)	13/83 (15.7)	4.8 (−5.3 to 14.9)	1.44 (0.67–3.11)	0.349	1.55 (0.73–3.30)	0.251
Second trimester pregnancy loss	5/92 (5.4)	2/83 (2.4)	−3.0 (−8.7–2.7)	0.44 (0.09–2.22)	0.448	0.35 (0.08–1.53)	0.163
Good birth outcome*	63/169 (37.3)	62/169 (36.7)	−0.6 (−10.9 to 9.7)	0.98 (0.75–1.30)	0.910	0.98 (0.75–1.29)	0.894

Data are n (%). RR, relative ratio. All multivariable models were adjusted for age, good-quality blastocyst transfer, preimplantation genetic testing cycle, antral follicle count, body mass index, and previous embryo transfer attempts.

* A good birth outcome was defined as a live birth at 37 weeks or more of gestation, with a birth weight between 2500 and 4000 g and without a major congenital anomaly.

*P *< 0.05 was considered statistically significant.

### Secondary outcomes

In the ITT population, the biochemical pregnancy rate was 62.1% in the day-6 progesterone group and 57.4% in the day-7 group (absolute difference, −4.7%; 95% CI, −15.2% to 5.7%; adjusted RR, 0.92 [95% CI, 0.78–1.09]; *P *= 0.315) (Table 2). The clinical pregnancy rate was 54.4% versus 49.1%, respectively (absolute difference, −5.3%; 95% CI, −16.0% to 5.3%; adjusted RR, 0.90 [95% CI, 0.74–1.09]; *P *= 0.282). Rates of total, biochemical, and clinical pregnancy loss were similar between groups. A good birth outcome occurred in 37.3% (63/169) of women in the day-6 group and 36.7% (62/169) in the day-7 group (absolute difference, −0.6%; 95% CI, −10.9% to 9.7%; adjusted RR, 0.98 [95% CI, 0.75–1.29]; *P *= 0.894; Table 2). No significant differences were observed between groups in obstetric or perinatal outcomes, including maternal complications (caesarean delivery, hypertensive disorders of pregnancy, gestational diabetes) and neonatal outcomes (preterm birth, birth weight, low birth weight, macrosomia, small or large for gestational age, major congenital anomalies, stillbirth) (Table 3). PP analyses yielded results consistent with the ITT analyses (Supplementary Tables S3 and S4).

**Table 3.** Obstetric and perinatal outcomes in the intention-to-treat population.

Outcomes	Day-6 progesterone group	Day-7 progesterone group	Absolute difference(95% CI)	Relative ratio(95% CI)	*P* value
	(n = 169)	(n = 169)			
Gestational age^#^	38.3 (2.1)	38.4 (1.1)	0.1 (−0.3 to 0.5)		0.592
Preterm birth^#^	6/77 (7.8)	4/68 (5.9)	−1.9 (−10.1 to 6.3)	0.75 (0.22–2.56)	0.750
Cesarean section^#^	52/77 (67.5)	50/68 (73.5)	6.0 (−8.8 to 20.8)	1.09 (0.88–1.34)	0.430
Hypertensive disorders of pregnancy^†^	6/92 (6.5)	7/83 (8.4)	1.9 (−5.9 to 9.7)	1.29 (0.45–3.69)	0.630
Gestational diabetes^†^	20/92 (21.7)	14/83 (16.9)	−4.9 (−16.5 to 6.8)	0.78 (0.42–1.44)	0.416
Birthweight (g)*	3234.5 (527.1)	3217.0 (380.6)	−17.5 (−167.1 to 132.1)		0.820
Singleton birthweight (g)	3234.5 (527.1)	3245.7 (370.8)	11.2 (−137.1 to 159.5)		0.886
Low birthweight*	3/76 (3.9)	0	−3.9 (−8.3 to 0.4)		0.246
Macrosomia*	5/76 (6.6)	2/70 (2.9)	−3.7 (−10.5 to 3.1)	0.43 (0.09–2.17)	0.444
Small for gestational age*	5/76 (6.6)	5/70 (7.1)	0.6 (−7.6 to 8.8)	1.09 (0.33–3.59)	1.000
Large for gestational age*	11/76 (14.5)	8/70 (11.4)	−3.0 (−13.9 to 7.8)	0.79 (0.34–1.85)	0.585
Major congenital anomaly^*^^∮^	0	1/70 (1.4)	1.4 (−1.4 to 4.2)		0.479
Stillbirth^#^	1/77 (1.3)	0	−1.3 (−3.8 to 1.2)		1.000

Data are n (%) or mean (SD). Continuous variables were compared using Mann–Whitney *U* test; categorical variables were compared using the chi-square test or Fisher’s exact test.

# Among all deliveries.

* Among live newborns.

† Among clinical pregnancies. *P *< 0.05 was considered statistically significant.

∮ There was one case of ventricular septal defect in the day-7 progesterone group.

### Post hoc analyses

**Figure 2.**
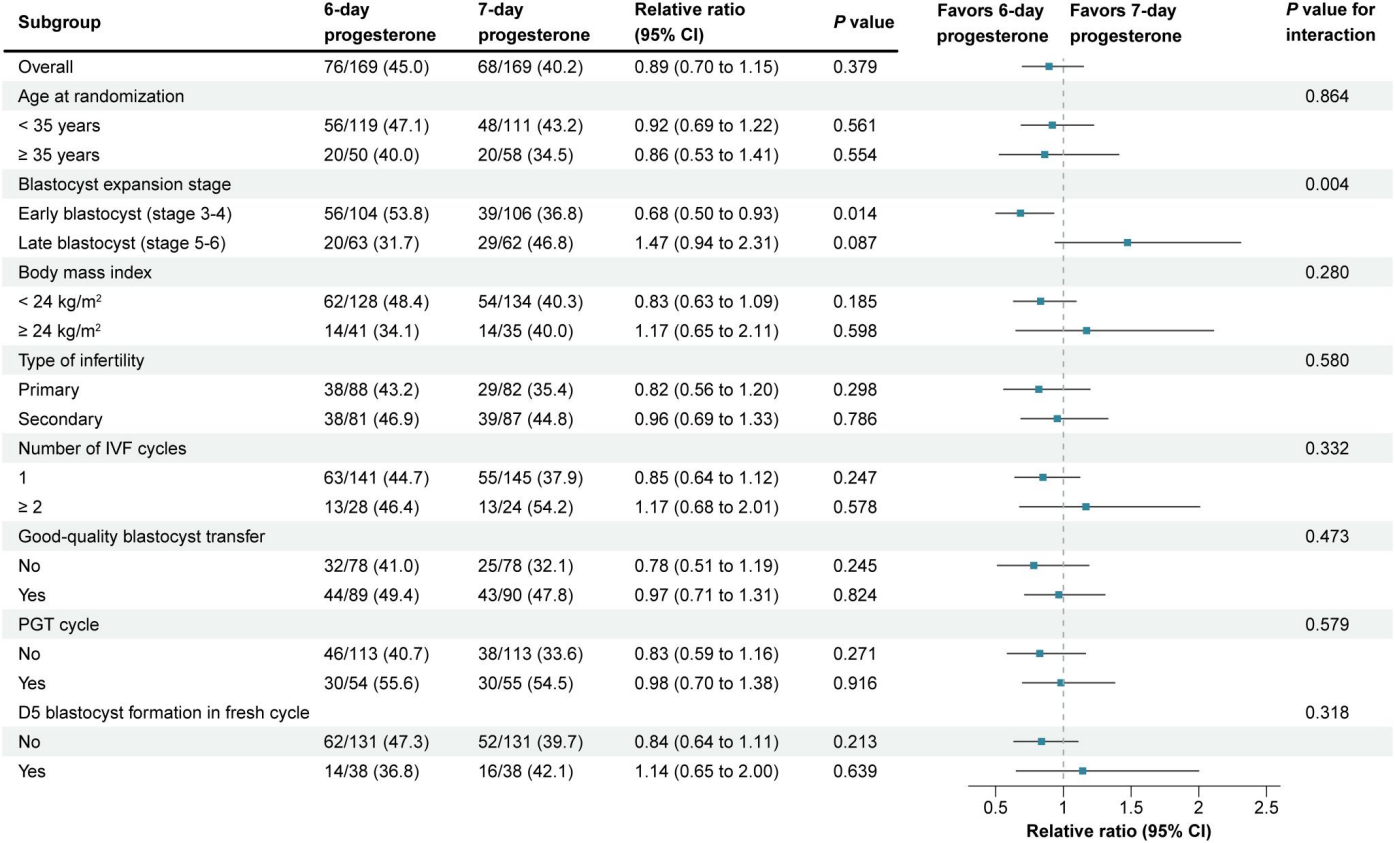
**Exploratory subgroup analyses for primary outcome (live birth rate) in intention-to-treat population**. PGT, preimplantation genetic testing; D5, day-5.

Subgroup analyses were performed in both the ITT and PP populations, stratified by age, blastocyst expansion stage, BMI, type of infertility, number of IVF cycles, good-quality blastocyst transfer, PGT cycle, and the presence of day-5 blastocyst formation in fresh cycle (Fig. 2; Supplementary Fig. S1). No significant interactions were observed between treatment group and these factors for LBR, with the exception of blastocyst expansion stage. In the ITT population, a significant interaction was identified between blastocyst expansion stage and treatment group (*P* for interaction = 0.004; Fig. 2), with consistent results observed when stratified by four expansion stages (*P* for interaction = 0.03) (Supplementary Table S5).

Among women receiving early blastocyst transfers (stage 3–4), the LBR was lower in the day-7 progesterone group compared with day-6 (36.8% vs 53.8%; absolute difference, −17.1%; 95% CI, −30.3% to −3.8%; adjusted RR, 0.69 [95% CI, 0.51–0.94]; *P *= 0.018; Supplementary Table S6). Conversely, for late blastocysts (stage 5–6), the LBR tended to be higher in the day-7 group (46.8% vs 31.7%; absolute difference, 15.0%; 95% CI, −1.9% to 32.0%; adjusted RR, 1.47 [95% CI, 0.95–2.28]; *P *= 0.082), though not statistically significant. For good birth outcomes, in the early blastocyst subgroup, rates were lower in the day-7 group than in the day-6 group (33.0% vs 45.2%; absolute difference, −12.2%; 95% CI, −25.3% to 0.9%; adjusted RR, 0.74 [95% CI, 0.53–1.04]; *P *= 0.086), whereas in the late blastocyst subgroup, rates were significantly higher in the day-7 group (43.5% vs 25.4%; absolute difference, 18.2%; 95% CI, 1.8–34.5%; adjusted RR, 1.72 [95% CI, 1.05–2.82]; *P *= 0.03) (Supplementary Table S6).

## Discussion

Based on this RCT, transferring day-6 blastocysts on the sixth or seventh day of progesterone supplementation resulted in comparable LBRs. A significant interaction was observed between blastocyst expansion stage and progesterone duration. Post hoc subgroup analyses indicated that among early blastocysts (stage 3–4), both clinical pregnancy and LBRs were significantly higher with the day-6 progesterone regimen. In contrast, among late blastocysts (stage 5–6), clinical pregnancy and LBRs did not differ significantly between groups, although both outcomes tended to favour the day-7 regimen; notably, the rate of good birth outcome was significantly higher in the day-7 group.

Evidence regarding the optimal timing of blastocyst transfer following progesterone priming in artificial cycles remains limited. Roelens *et al.* reported comparable overall LBRs between transfers performed on the sixth or seventh day of progesterone exposure, but observed a significantly higher miscarriage rate for day-6 blastocysts transferred on Day 6 compared with Day 7, while differences in LBR did not reach statistical significance (Roelens *et al.*, 2020). To date, only one RCT has examined progesterone duration prior to blastocyst transfer, reporting no significant difference in clinical pregnancy rates between transfers on the fifth versus seventh day of progesterone exposure (van de Vijver *et al.*, 2017). However, that study had a relatively small sample size, providing sufficient power to detect only a 16% absolute difference. Moreover, it allowed transfer of one or two blastocysts and did not account for the potential influence of the day of blastocyst cryopreservation on outcomes, both of which may have introduced bias.

Successful implantation relies on precise synchronization between embryo development and endometrial receptivity (Paria *et al.*, 2002). Although earlier studies suggested a relatively broad WOI following progesterone exposure, accumulating evidence indicates that the WOI is narrower than previously assumed (Theodorou and Forman, 2012; Franasiak *et al.*, 2016). Suboptimal progesterone priming may disrupt embryo–endometrium crosstalk, resulting in impaired implantation and adverse pregnancy outcomes (Wilcox *et al.*, 1999; Mackens *et al.*, 2017). These observations underscore the importance of aligning embryonic developmental stage with endometrial receptivity.

Embryos reaching the blastocyst stage on Day 6 have consistently been associated with lower implantation rates than those developing on Day 5 (Sunkara *et al.*, 2010; Haas *et al.*, 2016; Yang *et al.*, 2016; Ferreux *et al.*, 2018; Bourdon *et al.*, 2019), a finding often attributed to reduced intrinsic embryo quality (Sunkara *et al.*, 2010; Yang *et al.*, 2016). However, a potential mismatch between day-6 blastocysts and the WOI may also contribute (Franasiak *et al.*, 2016). Notably, most previous studies comparing day-5 and day-6 blastocysts performed embryo transfer uniformly on the sixth day of progesterone supplementation, leaving uncertainty as to whether ‘delayed’ day-6 blastocysts share the same optimal WOI as day-5 blastocysts.

Controlled ovarian stimulation (COS) is known to accelerate endometrial maturation and advance the WOI, and previous studies have shown improved outcomes for day-6 blastocysts transferred in frozen compared with fresh cycles (Shapiro *et al.*, 2014; Franasiak *et al.*, 2016). Nevertheless, few studies have specifically examined the optimal duration of progesterone exposure before FET for day-6 blastocysts, and existing evidence remains inconsistent. Existing studies can be broadly categorized into three groups: some have reported superior pregnancy outcomes with a day-7 progesterone regimen compared with a day-6 regimen (Roelens *et al.*, 2020; Zhou *et al.*, 2024); others have reported the opposite (Xu *et al.*, 2020; Bilgory *et al.*, 2021); while several studies have shown no significant differences between the two approaches (Qi *et al.*, 2024; Bhoi *et al.*, 2025). Importantly, these studies were retrospective, often did not distinguish between natural and artificial cycles, and did not account for blastocyst expansion stage.

Our trial—the first RCT focusing exclusively on women undergoing day-6 blastocyst transfer—demonstrated no significant difference in overall LBRs between six and seven days of progesterone supplementation. The observed interaction between blastocyst expansion stage and progesterone duration suggests that blastocysts at different developmental stages may require distinct lengths of progesterone priming. The opposing trends observed for early- and late-stage blastocysts may partly explain why both this trial and prior studies reported similar overall LBRs between progesterone regimens, as these effects may have counterbalanced each other at the population level.

Most previous studies have considered endometrial advancement induced by COS, progesterone duration, and blastocyst developmental timing as independent variables. Our findings highlight the importance of their dynamic interplay and suggest that progesterone timing for day-6 blastocysts may need to be individualized according to blastocyst expansion stage rather than applied uniformly. However, the study was not powered to formally evaluate post hoc subgroup analyses. Although baseline characteristics were balanced and multivariable analyses were performed, residual confounding cannot be excluded. These subgroup findings should therefore be interpreted as hypothesis-generating and require confirmation in adequately powered trials.

This study has several limitations. First, the open-label design may have introduced performance bias; however, embryologists were blinded, outcomes were objective, and baseline characteristics were well balanced, minimizing detection bias. Second, the study was not designed to evaluate differential effects across infertility subgroups. Although baseline infertility indications—including polycystic ovary syndrome, endometriosis, and repeated implantation failure—were well balanced between the two groups, subgroup-specific effects could not be assessed. Further studies are therefore warranted to determine whether the optimal timing of progesterone supplementation varies according to different infertility aetiologies. In addition, donor oocyte cycles were excluded because of their limited availability in our region, which may restrict generalizability to donor populations. Third, the trial was powered to detect a relatively large absolute difference in LBR (14%). While adequate for the primary hypothesis, it may have been underpowered to detect smaller but potentially clinically meaningful differences. The width of the 95% CI indicates that modest benefit or harm associated with either progesterone timing strategy cannot be excluded, and trials designed to detect smaller effects would require substantially larger sample sizes, which may be challenging in this population. Fourth, the luteal phase support regimen used in this study was relatively intensive and may not represent all HRT protocols currently in clinical practice. Higher progesterone exposure could theoretically accelerate endometrial maturation; however, individual endometrial receptivity was not directly assessed in this study, precluding evaluation of this potential effect. Although all participants received the same luteal phase support regimen, hereby preserving internal validity, the applicability of these findings may be limited to clinical settings using similar progesterone supplementation protocols. In addition, potential differences in oestradiol dosing strategies across centres should also be considered. Fifth, the LBR observed (∼40–45%) was relatively high, likely reflecting the inclusion of a favourable-prognosis population and strict eligibility criteria. As a single-centre study conducted in a high-volume tertiary centre, a centre effect may also have contributed, limiting external generalizability. Sixth, PGT cycles were not excluded from the study. Although the proportion of PGT cycles was balanced between groups and this factor was adjusted for in the analyses, residual confounding cannot be entirely excluded. Finally, the inclusion of two clinical contexts underlying the availability of only day-6 blastocysts may have introduced some degree of heterogeneity. Nevertheless, randomization and adjusted analyses mitigated major imbalances, and baseline characteristics serving as proxies for these contexts were well balanced between groups, suggesting that the primary findings are unlikely to be driven by embryo origin.

Future adequately powered RCTs are required to confirm these findings, especially the exploratory subgroup observations related to blastocyst expansion stage. In addition, studies incorporating progesterone monitoring or direct assessment of endometrial receptivity may help clarify the relationship between progesterone exposure, WOI dynamics, and reproductive outcomes.

## Supplementary Material

hoag023_Supplementary_Data

## Data Availability

Individual de-identified participant data supporting the findings of this study (including text, tables, figures, and appendices), along with the study protocol, will be made available. Data access will be granted to investigators whose proposed use of the data has been approved by an independent review committee established for this purpose. Data access requests should be submitted to liushine2006@163.com. The dataset will be accessible from 6 months to 5 years after publication through a third-party repository: http://www.medresman.org.cn/uc/projectsh/projectedit.aspx?proj=6245.
