# Supplementary material for: Effect of progesterone timing on live birth rates in day-6 blastocyst frozen-thawed embryo transfer cycles: a randomized controlled trial
Source: Hum Reprod Open. 2026 Mar 16;2026(2):hoag023. doi: 10.1093/hropen/hoag023 (PMC13020907; doi:10.1093/hropen/hoag023)
Supplement: hoag023_Supplementary_Data [file hoag023_supplementary_data.zip › Supplementary_Figure_S1-post adjudication clean.docx]

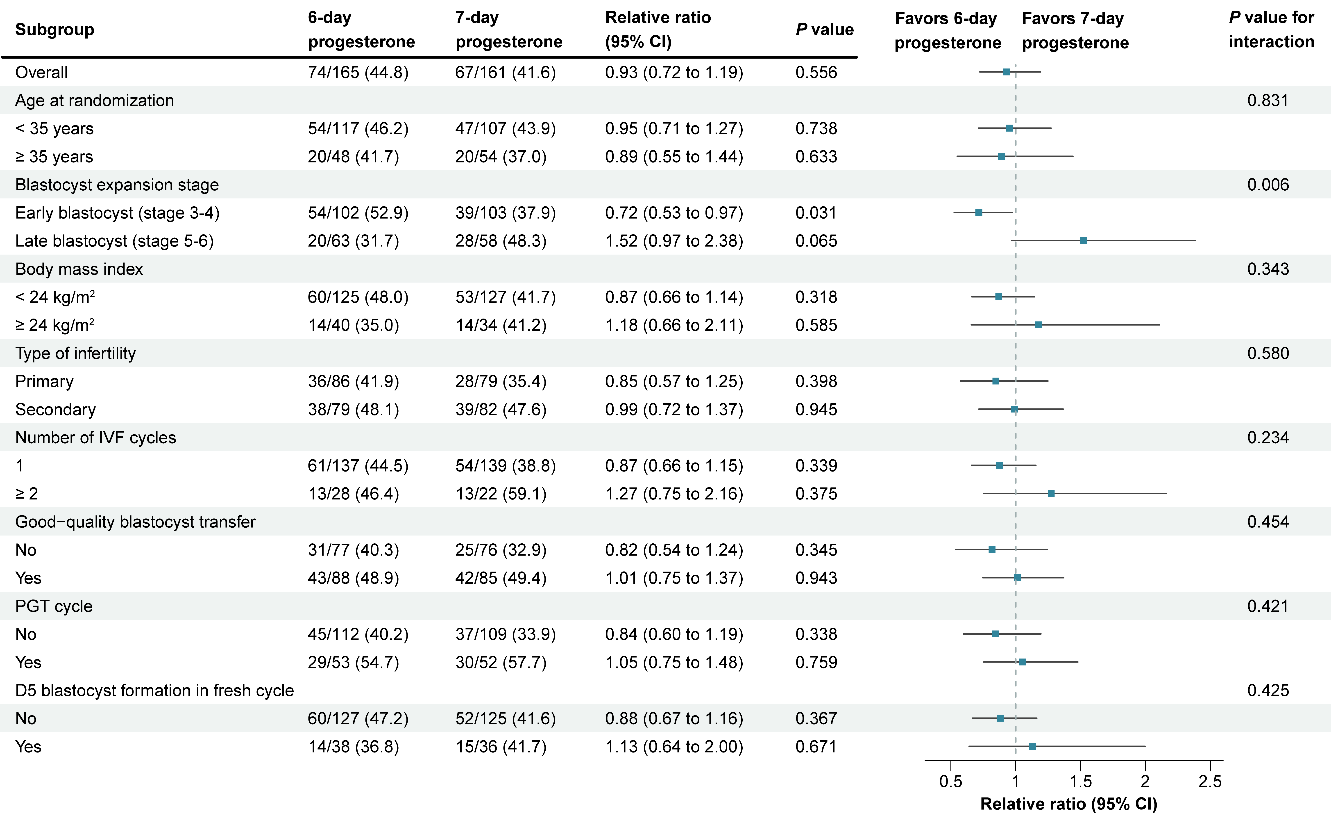


**Supplementary Figure S1.** Exploratory subgroup analyses for primary outcome in per-protocol population. PGT, preimplantation genetic testing; D5, day-5.
