# Supplementary material for: Effect of progesterone timing on live birth rates in day-6 blastocyst frozen-thawed embryo transfer cycles: a randomized controlled trial
Source: Hum Reprod Open. 2026 Mar 16;2026(2):hoag023. doi: 10.1093/hropen/hoag023 (PMC13020907; doi:10.1093/hropen/hoag023)
Supplement: hoag023_Supplementary_Data [file hoag023_supplementary_data.zip › Supplementary-Tables-post_adjudication_clean_EO.docx]

**Supplementary Table S1.** Definition of secondary outcomes.

| **Secondary outcomes** | **Definition** |
| --- | --- |
| Biochemical pregnancy | Positive serum β-hCG (≥10 IU/L) measured 12–15 days after embryo transfer. |
| Clinical pregnancy | Presence of an intrauterine gestational sac confirmed by ultrasound 28–35 days after embryo transfer. |
| Biochemical pregnancy loss | Initial positive β-hCG that did not progress to clinical pregnancy. |
| Clinical pregnancy loss | Clinical pregnancy not progressing to live birth. |
| Total pregnancy loss | Biochemical and clinical pregnancy loss. |
| Good birth outcome | A live birth at 37 weeks or more of gestation, with a birth weight between 2500 and 4000 g and without a major congenital anomaly. |
| Preterm birth | Delivery of a fetus at less than 37 and more than 24 weeks of gestation. |
| Hypertensive disorders of pregnancy | New-onset hypertension (systolic ≥140 mmHg and/or diastolic ≥90 mmHg measured at least twice, 4 hours apart) occurring after 20 weeks of gestation in woman with previously normal blood pressure. |
| Gestational diabetes | A condition characterized by varying degrees of glucose intolerance with onset or first diagnosis during pregnancy. |
| Birth weight | Neonatal body weight measured within 24 hours after birth. |
| Low birthweight | Birth weight < 2500 g |
| Macrosomia | Birth weight ≥ 4000 g |
| Small for gestational age | Birthweight < 10th percentile |
| Large for gestational age | Birthweight > 90th percentile |
| Major congenital anomalies | Major congenital anomalies were defined according to the criteria of the National Birth Defects Prevention Network and referred to structural malformations associated with significant medical, surgical, or cosmetic consequences, typically requiring clinical intervention. |
| Stillbirth | Fetus shows no signs of life at or after delivery. |

**Supplementary Table S2.** Baseline, IVF cycle, and FET cycle characteristics in the per-protocol population.

| **Parameters** | **Day-6 progesterone group**  **(n = 165)** | **Day-7 progesterone group**  **(n = 161)** |
| --- | --- | --- |
| **Baseline characteristics**  Age at retrieval (years)  Age at randomization (years) | 31.0 (28.0–34.0)  32.0 (30.0–35.0) | 31.0 (28.0–34.0)  32.0 (29.0–36.0) |
| Body mass index (kg/m^2^)  Duration of infertility (years)  Type of infertility  Primary  Secondary  Indications for IVF  Tubal factor  Male factor  Others  Combined factors  Unexplained | 21.6 (19.8–23.8)  2.0 (1.0–4.8)  86 (52.1)  79 (47.9)  60 (36.4)  29 (17.6)  53 (32.1)  13 (7.9)  10 (6.1) | 21.6 (19.9–23.6)  2.0 (1.0–4.0)  79 (49.1)  82 (50.9)  67 (41.6)  20 (12.4)  50 (31.1)  14 (8.7)  10 (6.2) |
| Antral follicle count  Polycystic ovary syndrome  Repeated implantation failure  Endometriosis  **IVF cycle characteristics**  Number of IVF cycles  1  2  3  ICSI treatment | 15 (10–22)  29 (17.6)  6 (3.6)  12 (7.3)  137 (83.0)  27 (16.4)  1 (0.6)  88 (53.3) | 16 (12–20)  30 (18.6)  4 (2.5)  11 (6.8)  139 (86.3)  22 (13.7)  0  86 (53.4) |
| Number of oocytes retrieved  Number of good-quality embryos on day 3  Number of blastocysts (D5 and D6)  Number of good-quality blastocysts (D5 and D6)  Without D5 blastocyst formation in fresh cycle  Number of D6 blastocysts  **FET cycle characteristics**  Previous embryo transfer attempts  Endometrial thickness*  Serum progesterone before progesterone^#^  Serum estradiol before progesterone^#^  Days of estradiol before progesterone^#^  Good-quality blastocyst transfer  PGT cycle  Blastocyst expansion stage  3  4  5  6 | 16 (12–22)  5 (3–7)  4 (2–5)  1 (0–2)  127 (77.0)  3 (2–5)  1 (0–1)  9.0 (8.0–10.3)  230.5 (175.6–295.1)  0.17 (0.09–0.24)  14 (13–17)  88 (53.3)  53 (32.1)  8 (4.8)  94 (57.0)  31 (18.8)  32 (19.4) | 17 (12–22)  5 (3–7)  4 (2–6)  1 (0–3)  125 (77.6)  3 (2–5)  1 (0–1)  9.0 (8.0–11.0)  230.5 (185.9–320.9)  0.17 (0.09–0.28)  15 (13–17)  85 (52.8)  52 (32.3)  6 (3.7)  97 (60.2)  33 (20.5)  25 (15.5) |

Data are median (interquartile range) or n (%). Continuous variables were compared using Mann–Whitney U test; categorical variables were compared using the chi-square test or Fisher’s exact test. FET, frozen embryo transfer; PGT, preimplantation genetic testing. *The day of frozen embryo transfer. ^#^The day before progesterone initiation. Repeated implantation failure was defined as failure to achieve a clinical pregnancy after ≥3 embryo transfer attempts, including the transfer of ≥2 good-quality blastocysts or ≥4 good-quality cleavage-stage embryos.

**Supplementary Table S3.** Per-protocol analysis for live birth, pregnancy, and pregnancy loss.

| **Outcomes** | **Day-6 progesterone group** | **Day-7 progesterone group** | **Absolute difference**  **(95% CI)** | **Unadjusted RR**  **(95% CI)** | **Unadjusted**  ***P* value** | **Adjusted RR**  **(95% CI)** | **Adjusted**  ***P* value** |
| --- | --- | --- | --- | --- | --- | --- | --- |
| Primary outcome  Total livebirth per women  Singleton livebirth per women  Twin livebirth per women | n = 165  74/165 (44.8)  74/165 (44.8)  0 | n = 161  67/161 (41.6)  66/161 (41.0)  1/161 (0.6) | −3.2 (−14.0 to 7.5)  −3.9 (−14.6 to 6.9)  0.6 (−0.6 to 1.8) | 0.93 (0.72 to 1.19)  0.91 (0.71 to 1.17) | 0.556  0.482  0.494 | 0.93 (0.73 to 1.18)  0.92 (0.72 to 1.17) | 0.555  0.483 |
| Biochemical pregnancy  Clinical pregnancy  Total pregnancy loss  Biochemical pregnancy loss  Clinical pregnancy loss  First trimester pregnancy loss  Second trimester pregnancy loss  Good birth outcome* | 103/165 (62.4)  90/165 (54.5)  28/103 (27.2)  13/103 (12.6)  15/90 (16.7)  10/90 (11.1)  5/90 (5.6)  61/165 (37.0) | 95/161 (59.0)  81/161 (50.3)  28/95 (29.5)  14/95 (14.7)  14/81 (17.3)  13/81 (16.0)  1/81 (1.2)  61/161 (37.9) | −3.4 (−14.0 to 7.2)  −4.2 (−15.1 to 6.6)  2.3 (−10.3 to 14.9)  2.1 (−7.5 to 11.7)  0.6 (−10.7 to 11.9)  4.9 (−5.4 to 15.2)  −4.3 (−9.6 to 1.0)  0.9 (−9.6 to 11.4) | 0.95 (0.79 to 1.13)  0.92 (0.75 to 1.13)  1.08 (0.70 to 1.69)  1.17 (0.58 to 2.35)  1.04 (0.53 to 2.01)  1.44 (0.67 to 3.11)  0.22 (0.03 to 1.86)  1.02 (0.77 to 1.36) | 0.527  0.444  0.721  0.665  0.914  0.345  0.214  0.864 | 0.94 (0.80 to 1.12)  0.92 (0.76 to 1.13)  1.09 (0.70 to 1.69)  1.17 (0.57 to 2.39)  1.05 (0.54 to 2.03)  1.57 (0.74 to 3.32)  0.17 (0.03 to 1.15)  1.03 (0.78 to 1.35) | 0.507  0.426  0.717  0.666  0.894  0.235  0.070  0.860 |

Data are n (%). RR, relative ratio. All multivariable models were adjusted for age, good-quality blastocyst transfer, preimplantation genetic testing cycle, antral follicle count, body mass index, previous embryo transfer attempts.

*A good birth outcome was defined as a live birth at 37 weeks or more of gestation, with a birth weight between 2500 and 4000 g and without a major congenital anomaly.

*P* < 0.05 was considered statistically significant.

**Supplementary Table S4.** Per-protocol analysis for obstetric and perinatal outcomes.

| **Outcomes** | **Day-6 progesterone group**  **(n = 165)** | **Day-7 progesterone group**  **(n = 161)** | **Absolute difference**  **(95% CI)** | **Relative ratio**  **(95%)** | ***P* value** |
| --- | --- | --- | --- | --- | --- |
| Gestational age^#^  Preterm birth^#^  Cesarean section^#^  Hypertensive disorders of pregnancy^†^  Gestational diabetes^†^  Birthweight (g)*  Singleton birthweight (g)  Low birthweight*  Macrosomia*  Small for gestational age*  Large for gestational age*  Major congenital anomalies*^∮^  Stillbirth^#^ | 38.3 (2.1)  6/75 (8.0)  51/75 (68.0)  6/90 (6.7)  20/90 (22.2)  3240.8 (531.8)  3240.8 (531.8)  3/74 (4.1)  5/74 (6.8)  5/74 (6.8)  11/74 (14.9)  0  1/75 (1.3) | 38.4 (1.1)  4/67 (6.0)  49/67 (73.1)  7/81 (8.6)  14/81 (17.3)  3226.7 (381.4)  3245.7 (370.8)  0  2/68 (2.9)  5/68 (7.4)  8/68 (11.8)  1/68 (1.5)  0 | 0.1 (−0.3 to 0.5)  −2.0 (−10.4 to 6.3)  5.1 (−9.8 to 20.1)  2.0 (−6.0 to 10.0)  −4.9 (−16.8 to 7.0)  −14.1 (−114.8 to 86.6)  4.9 (−94.8 to 104.6)  −4.1 (−8.5 to 0.4)  −3.8 (−10.8 to 3.2)  0.6 (−7.8 to 9.0)  −3.1 (−14.3 to 8.1)  1.5 (−1.4 to 4.3)  −1.3 (−3.9 to 1.3) | 0.75 (0.22 to 2.53)  1.08 (0.87 to 1.33)  1.30 (0.45 to 3.70)  0.78 (0.42 to 1.44)  0.44 (0.09 to 2.17)  1.09 (0.33 to 3.60)  0.79 (0.34 to 1.85) | 0.534  0.749  0.503  0.627  0.419  0.857  0.951  0.246  0.444  1.000  0.588  0.479  1.000 |

Data are n (%) or mean (SD). ^#^Among all deliveries. Continuous variables were compared using Mann–Whitney U test; categorical variables were compared using the chi-square test or Fisher’s exact test.

*Among live newborns. ^†^Among clinical pregnancies. *P* < 0.05 was considered statistically significant.

^∮^There was one case of ventricular septal defect in the day-7 progesterone group.

**Supplementary Table S5.** Live birth rates stratified by four blastocyst expansion stages (intention-to-treat population).

| **Subgroup** | **Day-6 progesterone group** | **Day-7 progesterone group** | **Relative ratio**  **(95% CI)** | ***P* value** | ***P* value for interaction** |
| --- | --- | --- | --- | --- | --- |
| **Blastocyst expansion stage**  Stage 3  Stage 4  Stage 5  Stage 6 | 6/9 (66.7)  50/95 (52.6)  8/31 (25.8)  12/32 (37.5) | 2/6 (33.3)  37/100 (37.0)  16/36 (44.4)  13/26 (50.0) | 0.50 (0.15 to 1.70)  0.70 (0.51 to 0.97)  1.72 (0.86 to 3.47)  1.33 (0.74 to 2.40) | 0.205  0.028  0.113  0.339 | 0.030 |

*P* < 0.05 was considered statistically significant.

**Supplementary Table S6.** Live birth, pregnancy, and pregnancy loss stratified by blastocyst expansion stage between the two treatment groups in the intention-to-treat population.

| **Outcomes** | **Day-6 progesterone group** | **Day-7 progesterone group** | **Absolute difference**  **(95% CI)** | **Unadjusted RR**  **(95% CI)** | **Unadjusted**  ***P* value** | **Adjusted RR**  **(95% CI)** | **Adjusted**  ***P* value** |
| --- | --- | --- | --- | --- | --- | --- | --- |
| **Early blastocyst (stage 3–4)**  Total livebirth per women  Singleton livebirth per women | N=104  56/104 (53.8)  56/104 (53.8) | N=106  39/106 (36.8)  39/106 (36.8) | −17.1 (−30.3 to −3.8)  −17.1 (−30.3 to −3.8) | 0.68 (0.50 to 0.93)  0.68 (0.50 to 0.93) | 0.014  0.014 | 0.69 (0.51 to 0.94)  0.69 (0.51 to 0.94) | 0.018  0.018 |
| Biochemical pregnancy  Clinical pregnancy  Total pregnancy loss  Biochemical pregnancy loss  Clinical pregnancy loss  Good birth outcome*  **Late blastocyst (stage 5–6)**  Total livebirth per women  Singleton livebirth per women  Biochemical pregnancy  Clinical pregnancy  Total pregnancy loss  Biochemical pregnancy loss  Clinical pregnancy loss  Good birth outcome* | 75/104 (72.1)  67/104 (64.4)  18/75 (24.0)  8/75 (10.7)  10/67 (14.9)  47/104 (45.2)  N=63  20/63 (31.7)  20/63 (31.7)  30/63 (47.6)  25/63 (39.7)  10/30 (33.3)  5/30 (16.7)  5/25 (20.0)  16/63 (25.4) | 58/106 (54.7)  48/106 (45.3)  19/58 (32.8)  10/58 (17.2)  9/48 (18.8)  35/106 (33.0)  N=62  29/62 (46.8)  27/62 (43.5)  39/62 (62.9)  35/62 (56.5)  10/39 (25.6)  4/39 (10.3)  6/35 (17.1)  27/62 (43.5) | −17.4 (−30.2 to −4.6)  −19.1 (−3.23 to −5.9)  8.8 (−6.7 to 24.2)  6.6 (−5.4 to 18.5)  3.8 (−10.1 to 17.8)  −12.2 (−25.3 to 0.9)  15.0 (−1.9 to 32.0)  11.8 (−5.1 to 28.7)  15.3 (−1.9 to 32.5)  16.8 (−0.5 to 34.0)  −7.7 (−29.4 to 14.0)  −6.4 (−22.8 to 10.0)  −2.9 (−22.9 to 17.2)  18.2 (1.8 to 34.5) | 0.76 (0.61 to 0.94)  0.70 (0.55 to 0.91)  1.36 (0.79 to 2.36)  1.62 (0.68 to 3.84)  1.26 (0.55 to 2.85)  0.73 (0.52 to 1.03)  1.47 (0.94 to 2.31)  1.37 (0.87 to 2.17)  1.32 (0.96 to 1.82)  1.42 (0.98 to 2.07)  0.77 (0.37 to 1.61)  0.62 (0.18 to 2.10)  0.86 (0.29 to 2.50)  1.71 (1.03 to 2.85) | 0.009  0.005  0.264  0.272  0.586  0.071  0.087  0.173  0.086  0.061  0.485  0.433  0.778  0.033 | 0.77 (0.63 to 0.94)  0.72 (0.56 to 0.92)  1.37 (0.78 to 2.38)  1.72 (0.71 to 4.16)  1.17 (0.50 to 2.73)  0.74 (0.53 to 1.04)  1.47 (0.95 to 2.28)  1.36 (0.87 to 2.13)  1.33 (0.97 to 1.81)  1.42 (1.00 to 2.02)  0.75 (0.36 to 1.55)  0.69 (0.20 to 2.41)  0.84 (0.32 to 2.19)  1.72 (1.05 to 2.82) | 0.012  0.008  0.272  0.232  0.716  0.086  0.082  0.176  0.073  0.048  0.437  0.561  0.720  0.030 |

Data are n (%). RR, relative ratio. All multivariable models were adjusted for age, good-quality blastocyst transfer, preimplantation genetic testing cycle, antral follicle count, body mass index, and previous embryo transfer attempts.

*P* < 0.05 was considered statistically significant. *A good birth outcome was defined as a live birth at 37 weeks or more of gestation, with a birth weight between 2500 and 4000 g and without a major congenital anomaly.
